# Safety of rFVIIa in hemodynamically unstable polytrauma patients with traumatic brain injury: *post hoc *analysis of 30 patients from a prospective, randomized, placebo-controlled, double-blind clinical trial

**DOI:** 10.1186/cc6092

**Published:** 2007-08-08

**Authors:** Yoram Kluger, Bruno Riou, Rolf Rossaint, Sandro B Rizoli, Kenneth David Boffard, Philip Iau Tsau Choong, Brian Warren, Michael Tillinger

**Affiliations:** 1 Department of Surgery, Rambam Medical Center, POB 9602, Haifa 31096, Israel; 2 Departments of Emergency Medicine and Surgery and Anesthesiology and Critical Care, Hôpital Pitié Salpêtrière, Assistance Publique-Hôpitaux de Paris, Université Pierre et Marie Curie-Paris, Paris, France; 3 Institute for Anesthesiology, University Clinics, Aachen, Germany; 4 Sunnybrook Health Sciences Centre, University of Toronto, Toronto, ON, Canada; 5 Department of Surgery, Johannesburg Hospital, Johannesburg, South Africa; 6 National University Hospital, Singapore; 7 Department of Surgery, University of Stellenbosch, Tygerberg, South Africa; 8 Novo Nordisk A/S, Bagsværd, Denmark

## Abstract

**Background:**

Trauma is a leading cause of mortality and morbidity, with traumatic brain injury (TBI) and uncontrolled hemorrhage responsible for the majority of these deaths. Recombinant activated factor VIIa (rFVIIa) is being investigated as an adjunctive hemostatic treatment for bleeding refractory to conventional replacement therapy in trauma patients. TBI is a common component of polytrauma injuries. However, the combination of TBI with polytrauma injuries is associated with specific risk factors and treatment modalities somewhat different from those of polytrauma without TBI. Although rFVIIa treatment may offer added potential benefit for patients with combined TBI and polytrauma, its safety in this population has not yet been assessed. We conducted a *post hoc *sub analysis of patients with TBI and severe blunt polytrauma enrolled into a prospective, international, double-blind, randomized, placebo-controlled study.

**Methods:**

A *post hoc *analysis of study data was performed for 143 patients with severe blunt trauma enrolled in a prospective, randomized, placebo-controlled study, evaluating the safety and efficacy of intravenous rFVIIa (200 + 100 + 100 μg/kg) or placebo, to identify patients with a computed tomography (CT) diagnosis of TBI. The incidences of ventilator-free days, intensive care unit-free days, and thromboembolic, serious, and adverse events within the 30-day study period were assessed in this cohort.

**Results:**

Thirty polytrauma patients (placebo, *n *= 13; rFVIIa, *n *= 17) were identified as having TBI on CT. No significant differences in rates of mortality (placebo, *n *= 6, 46%, 90% confidence interval (CI): 22% to 71%; rFVIIa, *n *= 5, 29%, 90% CI: 12% to 56%; *P *= 0.19), in median numbers of intensive care unit-free days (placebo = 0, rFVIIa = 3; *P *= 0.26) or ventilator-free days (placebo = 0, rFVIIa = 10; *P *= 0.19), or in rates of thromboembolic adverse events (placebo, 15%, 90% CI: 3% to 51%; rFVIIa, 0%, 90% CI: 0% to 53%; *P *= 0.18) or serious adverse events (placebo, 92%, 90% CI: 68% to 98%; rFVIIa, 82%, 90% CI: 60% to 92%; *P *= 0.61) were observed between treatment groups.

**Conclusion:**

The use of a total dose of 400 (200 + 100 + 100) μg/kg rFVIIa in this group of hemodynamically unstable polytrauma patients with TBI was not associated with an increased risk of mortality or with thromboembolic or adverse events.

## Introduction

Trauma is the leading cause of mortality and severe morbidity among young adults (15 to 44 years of age), with traumatic brain injury (TBI) and uncontrolled bleeding responsible for the majority of these deaths [1-3]. Although some progress has been made in managing traumatically induced surgical bleeding, treatment of the multifactorial coagulopathic component of traumatic hemorrhage remains a serious clinical challenge. Hence, uncontrolled bleeding constitutes a leading cause of in-hospital mortality despite adequate replacement therapy with fresh frozen plasma (FFP), platelets, cryoprecipitate, and fibrinogen [4-7]. Recombinant activated factor VII (rFVIIa) has been reported as a possible adjunctive, 'off label' treatment for coagulopathic bleeding that is refractory to conventional replacement therapy in a growing number of case series and reports, with several expert-opinion guidelines now published [8-15].

The results of the first prospective, multicenter, randomized, placebo-controlled studies of rFVIIa in blunt and penetrating trauma have been published recently [16]. The incidence of adverse events (AEs), thromboembolic (TE) events, and serious adverse events (SAEs) was evenly distributed between treatment groups, and no safety concerns for the use of rFVIIa in these patients were raised.

TBI is a common component of the polytrauma injury complex, especially among patients with blunt trauma [17]. Patients sustaining combined TBI with polytrauma constitute a special subpopulation. These patients typically have a poorer prognosis [17] and a higher risk for developing coagulopathy and TE events and require different treatment considerations. For instance, permissive hypotension is not recommended for TBI [18].

Theoretically, rFVIIa may be of particular added benefit for patients with polytrauma and TBI. As adequate cerebral perfusion pressure is an important goal of treatment to prevent secondary brain insult [19,20], arresting bleeding and maintaining hemodynamic stability are of even greater importance in hemodynamically unstable patients with TBI. In addition, rFVIIa may prevent the expansion of traumatic intracerebral hemorrhage (ICH) in a manner similar to that demonstrated by the recently published controlled study of spontaneous ICH patients [21] and as reported by a number of case series [8,22,23].

Despite these potential advantages and the relative success and safe profile of rFVIIa described in several case series of isolated TBI and other central nervous system (CNS) bleedings [8,22-31], there is relatively little clinical experience and therefore very limited safety evaluation of rFVIIa use in patients with combined TBI and polytrauma injuries [8]. In addition, some safety concerns, specifically regarding TE events, have arisen following the use of rFVIIa in CNS bleeding [21,32,33]. There are also some theoretical concerns of a possible excessive activation of the clotting system with rFVIIa in such injuries, due to the release of tissue factor (TF) in the brain and the prevalence of consumption coagulopathy or disseminated intravascular coagulation in brain injuries [34,35].

To assess the safety of rFVIIa in polytrauma with TBI, we have analyzed the safety data for severely injured blunt-trauma patients who were included in a prospective, international, double-blind, randomized, placebo-controlled study of rFVIIa [16] and who were diagnosed by the investigators by computed tomography (CT) to have had TBI.

## Materials and methods

The methods of the placebo-controlled study have been reported previously [16]. In brief, patients were evaluated for inclusion in the trial on admission to the trauma center. Inclusion criteria included receipt of 6 units of red blood cells (RBCs) within a 4-hour period and known age of between 16 (or legally of age, according to local law) and 65 years. Main exclusion criteria were cardiac arrest prehospital or in the emergency or operating room prior to trial drug administration; gunshot wound to the head; base deficit of greater than 15 mEq/l or severe acidosis with pH of less than 7.00; transfusion of 8 or more units of RBCs prior to arrival at the trauma center; injury sustained greater than or equal to 12 hours before randomization; and severe TBI, defined as a Glasgow Coma Scale (GCS) score of less than or equal to 8, unless in the presence of a normal head CT scan. The protocol for the placebo-controlled study was approved by the ethics committee of each participating institution, and the trial was conducted according to Good Clinical Practice standards, with appropriate informed consent, as described previously [16].

Eligible patients were randomly assigned to treatment groups after receiving 6 units of RBCs within a 4-hour period. Treatment arms were either three intravenous injections of rFVIIa (200, 100, and 100 μg/kg; NovoSeven^®^; Novo Nordisk A/S, Bagsværd, Denmark) or three placebo injections. The first dose of study drug was administered immediately after transfusion of the eighth unit of RBCs given that the patient, in the opinion of the attending physician, would require additional transfusions. The second and third doses followed 1 and 3 hours after the first dose, respectively. Study drug was administered in addition to standard treatment for injuries and bleeding at the participating hospitals.

### Traumatic brain injury *post hoc *subanalysis

In accordance with protocol inclusion criteria, all patients were hemodynamically unstable (6 units of RBCs within 4 hours of admission and ongoing bleeding as determined by the investigator). Treatment priorities in such hemodynamically unstable patients preclude any clinical or ethical possibility of performing a prospective baseline head CT, which would be required for an accurate diagnosis and severity assessment of the head injury in the majority of these patients.

Therefore, to identify patients with a TBI component of their injury, we were obliged to perform a *post hoc *subanalysis. This analysis was based on CT imaging findings, which were obtained at the investigator's clinical judgment, after enrollment, and only upon reaching clinical stabilization of the patients.

The severity of TBI was prospectively assessed by both the GCS and Abbreviated Injury Score (AIS). However, for the purposes of identifying patients with TBI for this analysis, only the AIS (as reported by investigators) was used for screening. This is because the AIS is based on the objective anatomical findings on CT imaging and also because the accuracy of the GCS assessment is limited in ventilated or pharmacologically paralyzed patients, such as those enrolled into this analysis.

All data for patients with AIS of any severity (1 to 6) in the anatomical region of the head (region 1) were reviewed manually by a physician who was blinded to the therapy arm. Patients who met the criteria of descriptors of injury that fit accepted definitions of TBI were included in this analysis. The incidences of AEs, SAEs, TE events, ventilator-free days, and intensive care unit (ICU)-free days were evaluated over the study period of 30 days.

### Statistical analyses

Data are expressed as mean ± standard deviation, medians [minimum-maximum], and percentages with their 90% confidence interval (CI). Comparison of two means was performed using the Student *t *test, comparison of two medians using the Wilcoxon test, and comparison of two proportions using the Fisher exact test. All *P *values were two-tailed, and a *P *value of less than 0.05 was considered significant.

## Results

Of the 143 blunt polytrauma patients randomly assigned into the prospective trial [16], a total of 30 (21%) patients were identified as having a TBI component. The main TBI diagnoses on CT were subarachnoid hemorrhage, occurring in 10 of 30 (33%) patients; intracerebral contusion or hematoma, occurring in 10 of 30 (33%) patients; and other types of TBI (two subdural hemorrhages, two depressed fractures, one diffuse axonal injury, one ischemia, one edema, one intraventricular hemorrhage, and two unspecified), occurring in the remaining 10 of 30 (33%) patients with TBI. Thirteen (43%) of the patients with TBI were in the placebo group, and 17 (57%) were in the rFVIIa group. Despite the fact that enrollment was based on the severity of bleeding caused by the systemic polytrauma rather than the TBI component of the injury, baseline characteristics and severity of TBI were similar for patients in the placebo and treatment groups (Table 1).

**Table 1:** Patient characteristics: baseline parameters

	Placebo (*n *= 13)	rFVIIa (*n *= 17)
Female, number (percentage)	5 (38)	7 (41)
Age, years	32.6 ± 16.8	33.5 ± 13.7
Injury Severity Score	36.8 ± 12.8	38.7 ± 13.7
Abbreviated Injury Score head (region 1)	3 [3-5]	3 [3-5]
Mean arterial pressure, mm Hg	76 ± 22	71 ± 18 (*n *= 16)
Body temperature, °C	35.1 ± 1.3 (*n *= 8)	34.3 ± 1.8 (*n *= 9)
pH	7.24 ± 0.12 (*n *= 11)	7.22 ± 0.10 (*n *= 15)

Baseline refers to predosing. All data are presented as number of patients (percentage) or mean (± standard deviation shown in most cases) or median [minimum-maximum shown], and *n *is indicated in cases in which there are missing values. rFVIIa, recombinant activated factor VII.

### Safety assessment

#### Mortality

The results of the safety assessment are presented in Table 2. A total of 11 of 30 (37%) patients died during the 30-day follow-up: 6 of 13 (46%; 90% CI, 22% to 71%) in the placebo group and 5 of 17 (29%; 90% CI, 12% to 56%) in the rFVIIa group (*P *= 0.19) (Table 2).

**Table 2:** Comparison of safety parameters between placebo- and rFVIIa-treated patients

	Placebo	rFVIIa	*P *value
Number of patients	13	17	-
Adverse events	12 (92; 68–98)	15 (88; 67–96)	1
Patients	31	44	
Events			
Serious adverse events			
Patients	12 (92; 68–98)	14 (82; 60–92)	0.61
Events	26	33	
Thromboembolic serious adverse events^a^			
Patients	2 (15; 3–51)	0 (0; 0–53)	0.18
Events	2	0	
Mortality (total)	6 (46; 22–71)	5 (29; 12–56)	0.19
Early mortality (≤48 hours)	3 (23; 7–56)	2 (12; 2–43)	0.63
Late mortality (>48 hours to 30 days)	3 (23; 7–56)	3 (18; 5–47)	1
Multiorgan failure	2 (15; 3–51)	3 (18; 5–47)	1
Acute respiratory distress syndrome	2 (15; 3–51)	2 (12; 2–43)	1
Intensive care unit-free days^b^	0 [0–21]	3 [0–23]	0.26
Ventilator-free days^b^	0 [0–25]	10 [0–24]	0.19

Data are presented as number of patients (percentage; 90% confidence interval) or median [minimum-maximum]. ^a^Both thromboembolic serious adverse events were part of the entire cohort of 12 serious adverse events reported for the placebo group. ^b^*P *values apply to the two-sided Wilcoxon rank test. All other *P *values apply to the two-sided Fisher exact tests. rFVIIa, recombinant activated factor VII.

Early mortality (less than or equal to 48 hours) was encountered by 3 of 13 (23%; 90% CI, 7% to 56%) patients in the placebo group: one death from cardiac contusion within 3 hours of hospital admission, one death from hypovolemic shock within 5 hours of hospital admission, and one death from TBI (right middle cerebral artery infarct) within 44 hours after hospital admission.

Similarly, there were 2 of 17 (12%; 90% CI, 2% to 43%) early mortalities reported in the rFVIIa group. Both of these deaths occurred as a result of hypovolemic shock: one within 5 hours of hospital admission and the other within 17 hours of hospitalization. Therefore, there was no difference in the rate of early mortality between placebo- and rFVIIa-treated patients (*P *= 0.63).

Late mortality (within 30 days) was encountered by 3 of 13 (23%; 90% CI, 7% to 56%) patients in the placebo group: one from brain death 3 days (54 hours) after hospital admission, one from multiorgan failure (MOF) 5 days (125 hours) after hospital admission, and one from pulmonary embolism, confirmed by postmortem, 5 days (114 hours) after hospitalization.

In the rFVIIa group, there were 3 of 17 (18%; 90% CI, 5% to 47%) late mortalities, one of which was from persistent elevated intracranial pressure (ICP) despite two surgical interventions and extensive medical and pharmacological treatment. The patient died 8 days (188 hours) after admission. Another death was caused by MOF, confirmed by postmortem, within 3 days (58 hours) of hospital admission. The third death was caused by sepsis 11 days (270 hours) after hospitalization. There was no difference in the rate of late deaths between placebo- and rFVIIa-treated patients (*P *= 1.00).

#### Serious adverse events and thromboembolic events

There were no significant differences in the incidence of reported SAEs and TE events for the two groups. SAEs were reported for 12 patients (92%) who had received placebo and 14 patients (82%) who had received rFVIIa (*P *= 0.61) (Table 2). Of these SAEs, there were 2 of 13 (15%; 90% CI, 3% to 51%) TE SAEs reported in the placebo group; one was a fatal pulmonary embolism and the other a subclavian vein thrombosis that was resolved with treatment. There were no TE AEs (0%; 90% CI, 0% to 53%) in the rFVIIa group (*P *= 0.18). There were no significant differences in the number of patients who experienced MOF and acute respiratory distress syndrome (ARDS) or in the number of ICU-free days or ventilator-free days (Table 2).

## Discussion

### Clinical use of recombinant activated factor VIIa

#### General

rFVIIa (NovoSeven^®^; Novo Nordisk A/S) is indicated for the treatment of bleeding episodes and for the prevention of bleeding during surgery or invasive procedures in patients with congenital hemophilia A and B with inhibitors to coagulation factors VIII (FVIII) or IX (FIX) or in those expected to have a high anamnestic response to FVIII or FIX, acquired hemophilia, congenital FVII deficiency, and in Europe for Glanzmann's thrombasthenia refractory to platelet transfusions.

Since the first report of the successful use of rFVIIa in an Israeli patient with a penetrating gunshot wound to the vena cava in 1999 [36], there has been an increasing number of case reports and series describing the 'off label' treatment of coagulopathic bleeding in a wide array of clinical scenarios. These publications have described hematological indications [37], reversal of anticoagulation [38,39], as well as bleeds in critically ill patients, such as in civilian and military trauma [8-10,40], cardiac surgery [41], postpartum hemorrhage [42,43], and other clinical situations in which impaired hemostasis has posed a serious, and often life-threatening, therapeutic challenge. A thorough review of these uses is beyond the scope of this paper and can be found elsewhere [44,45].

#### Central nervous system bleeds

The clinical use of rFVIIa in CNS bleeds has also been published. Bleeding in these patients resulted from a variety of etiologies, including TBI, spontaneous ICH, neurosurgery, anticoagulation medications, and underlying hematological disease [8,21-31,46].

#### Traumatic brain injury

Dutton and colleagues [8] described a series of 81 coagulopathic trauma patients treated with rFVIIa. Of these, 20 received rFVIIa for treatment of coagulopathy related to TBI. Six of these patients had additional polytrauma. The outcome of these patients was poor and 15 of 20 patients died. The authors attributed this high mortality rate to the severity of brain injury. None of the 81 trauma patients in this series had any clinical indication of TE events.

Zaaroor and Bar-Lavie [23] reported the first series of five patients with TBI with a hemorrhagic component in whom rFVIIa treatment was reported to be effective in controlling the evolution of intracerebral brain contusion and bleeding. Four patients presented with a penetrating head injury, and one with a blunt head injury. In all patients, hemorrhagic brain contusion was encountered with the potential for expansion that could have led to severe neurological deterioration as deemed by the authors. Limited expansion was noted subsequent to treatment with 90 to 100 μg/kg rFVIIa, and no TE AEs were attributed to administration of this agent.

Morenski and colleagues [24] described the use of 90 μg/kg rFVIIa in three pediatric TBI cases in which coagulopathy prevented the insertion of an ICP monitor, which was deemed crucial for guiding optimal treatment. The youngest patient was 5 weeks old. In all three patients, coagulopathy persisted despite treatment with FFP. Administration of rFVIIa corrected the coagulopathy, allowing for the successful insertion of the ICP monitor with no TE events observed.

### Safety of recombinant activated factor VIIa

Overall, rFVIIa is considered to have a favorable safety profile in hemophilia and in critical bleedings across a broad array of clinical scenarios [47-51]. However, because of its prohemostatic activities, concerns persist over the risk for TE events during its clinical use [52].

The previously mentioned randomized, controlled studies in blunt and penetrating trauma [16], which forms the basis for this analysis, have demonstrated no safety concerns when using rFVIIa in trauma patients. Thus, TE events occurred in 4% (6 of 138) of the placebo-treated patients as compared with 4% (6 of 139) of the rFVIIa-treated patients. The incidence of fatal TE events was low and did not differ between the treatments groups (1% in the placebo group versus 1% in the rFVIIa group) [16,49].

In a recent review based on 13 different controlled clinical trials in which rFVIIa has been studied in patients with coagulopathy secondary to the use of anticoagulant therapy, cirrhosis, or severe trauma (including a detailed safety profile of the study by Boffard and colleagues [16] described within this paper), it was found that there was no significant difference between placebo-treated and rFVIIa-treated patients with respect to TE AEs, either in the individual trials or when the study populations were combined (5.3% (23 of 430) of placebo-treated patients and 6.0% (45 of 748) of rFVIIa-treated patients; (*P *= 0.57) [49].

This safety profile can probably be attributed to the localized activation of coagulation at the site of injury [49-51]. At pharmacological doses, rFVIIa induces hemostasis by binding either to TF or directly to activated platelets, which are the physiological markers of tissue injury. This initiates a cascade that results in a thrombin burst and the formation of a stable fibrin plug [37,53].

Despite this encouraging safety profile, several publications regarding the use of rFVIIa in CNS bleeding have raised some safety concerns:

In a recently published controlled study of rFVIIa in spontaneous ICH [21], 399 patients received placebo or 40, 80, or 160 μg/kg rFVIIa. A significant reduction in hematoma size, mortality, and morbidity was observed in the rFVIIa-treated group. TE AEs, mainly myocardial or cerebral infarction, occurred in 7% of the rFVIIa-treated patients compared with 2% in the placebo group (*P *= 0.12). There were no arterial TE SAEs in the placebo group; the overall frequency of such events was 5% among the rFVIIa-treated patients (*P *= 0.01 by Fisher exact test). However, TE SAEs that were possibly or probably related to treatment and that were fatal or disabling occurred equally (2%) in the rFVIIa-treated group and the placebo group. An ongoing phase III study is likely to provide a better evaluation of safety in this patient population.

Pickard and colleagues [33] conducted an open-label, dose-escalation safety study of rFVIIa in the prevention of re-bleeding following aneurysmal subarachnoid hemorrhage. The trial was designed to include 15 patients who would be treated with either a single bolus of 80 μg/kg rFVIIa or a bolus of 80 μg/kg followed by a continuous infusion at either 3.5 or 7 μg/kg per hour compared with controls. The 10th consecutive enrolled patient developed a middle cerebral artery branch thrombosis contralateral to the aneurysm. This patient had received the 80 μg/kg bolus of rFVIIa followed by a continuous infusion of 7 μg/kg per hour. He developed hemiparesis ipsilateral to the aneurysm on day 4, approximately 2.5 hours after the rFVIIa treatment was stopped. The study was discontinued as a result of this thrombotic event despite the higher incidence of thrombotic events reported for the overall subarachnoid hemorrhage population [54].

Siegel and colleagues [32] reported on a 19-year-old polytrauma patient suffering from an open shaft fracture of the femur, pneumothorax, lung contusion, and a mild TBI (GCS = 15) with no intracranial pathology on initial CT. The patient was treated with 60 μg/kg rFVIIa to control bleeding from his thigh 12 hours after orthopedic surgery for stabilizing his fracture. The patient was on prolonged ventilation due to his lung contusion. Upon the achievement of spontaneous ventilation, there were changes in his level of consciousness. A CT performed on day 5 revealed a small frontal contusion. On day 21, after a complicated neurological work-up, the patient was diagnosed with a cerebral sinus thrombosis, from which he gradually recovered. The authors concluded that due to the short half-life of rFVIIa, a direct relationship between rFVIIa and the thrombus was unlikely, but they could not completely rule out a possible correlation.

Thomas and colleagues [55] have retrospectively reviewed TE events in 285 patients who received rFVIIa for a variety of clinical indications in their institution from 2001 to 2006. Most patients were treated with rFVIIa for acute hemorrhagic shock (*n *= 142; 50%), TBI (*n *= 100; 33%), and reversal of warfarin therapy (*n *= 7; 2%). Twenty-seven patients (9.4%) had TE complications, and nine of these events (3.1%) were thought by a panel of experts evaluating causality retrospectively to be highly related to rFVIIa. Eighteen of the TE events were attributed to a combination of rFVIIa and a definable, high-energy vascular injury. The authors noted that in addition to the subjectivity of their assessment, the time gap (>24 hours) between rFVIIa administration and the majority of TE complications hindered their ability to ascertain a relationship with the short-acting rFVIIa, especially in the high-risk trauma population and without the benefit of a control group to allow comparisons. They recommend earlier surveillance for TE complications and the publication of 'off label' experience from large trauma centers.

Additional safety concerns were raised by O'Connell and colleagues [53], who recently reviewed 168 spontaneous reports that were sent to the U.S. Food and Drug Administration concerning TE events, of which 151 occurred in 'off label' clinical use in adults and children. Although such events were relatively uncommon, they often resulted in serious morbidity and mortality. The analysis of the relationship between AEs and rFVIIa was hindered by concomitant medications and pre-existing medical conditions and was confounded by various indications and the inherent limitations of passive surveillance. They concluded that randomized, controlled trials are needed to establish the safety and efficacy of rFVIIa in patients without hemophilia.

We report the first safety data collected in the setting of a randomized, controlled study for patients sustaining TBI with polytrauma. Our results showed no significant differences in mortality, TE AEs, SAEs or AEs, ARDS, MOF, ICU-free days, or ventilator-free days between the rFVIIa and placebo groups. It is important to note that the safety profile demonstrated in this subanalysis was achieved despite the administration of a significantly higher rFVIIa dose regime (200 + 100 + 100 μg/kg) in comparison with all previously reported case series, in which the dosage of rFVIIa used in both trauma or CNS bleedings ranged from 16 to 120 μg/kg.

### Study limitations

Our study has some inherent limitations. Our findings are based on a subgroup analysis with a sample size that was not powered to exclude a safety signal between the two treatment arms. Indeed, the randomized, controlled trauma study was designed specifically to exclude severe TBI in order to avoid adding heterogeneity to the already heterogeneous trauma population. Nevertheless, it should be noted that the safety profile for rFVIIa in patients with TBI and polytrauma injuries is similar in nature to that of the entire cohort of 277 polytrauma patients, in whom no safety differences were found between those treated with rFVIIa and with placebo [16]. A larger phase III study in polytrauma which allows for the inclusion of a subgroup of patients with TBI is ongoing and is likely to provide additional safety data for the subgroup of patients with TBI and polytrauma.

Another limitation of our analysis is the lack of any data concerning the effect of rFVIIa on the actual TBI. This inherent limitation of our study stems from the hemodynamic instability of these injuries. This predicament precludes any clinical or ethical possibility of obtaining baseline and periodically repeated head CT imaging in a timely fashion, which would be required to evaluate any significant clinical data on the course of the head injury itself and on the potential safety and efficacy of rFVIIa in the treatment of this type of injury.

Although the present study adds to our ability to assess safety with regard to rFVIIa and TBI, more information is needed. Data concerning the safety and possible efficacy of rFVIIa in patients with polytrauma and TBI will need to be deduced from studies in hemodynamically stable patients with TBI. A dose-escalation study aimed primarily at assessing the safety of rFVIIa in TBI has recently been completed, and data analysis is ongoing.

## Conclusion

The use of rFVIIa in this subgroup of hemodynamically unstable patients suffering from blunt polytrauma with TBI injuries was not associated with an increased risk of mortality, TE events, or SAEs. Ongoing studies will provide additional data to improve the safety assessment of rFVIIa.

## Key messages

• Traumatic brain injury (TBI) and uncontrolled hemorrhage are responsible for the majority of trauma deaths.

• Recombinant activated factor VIIa (rFVIIa) is being investigated as an adjunctive hemostatic treatment for bleeding refractory to conventional therapy in trauma patients.

• Although rFVIIa treatment may offer added potential benefit for patients with combined TBI and polytrauma, its safety in this population has not yet been assessed.

• A *post hoc *analysis was performed for 143 patients with severe blunt trauma enrolled in a prospective, randomized, placebo-controlled study, evaluating the safety and efficacy of intravenous rFVIIa (200 + 100 + 100 μg/kg) or placebo to identify patients with a computed tomography diagnosis of TBI.

• No significant differences in rates of mortality, themedian numbers of intensive care unit-free days or ventilator-free days, or rates of thromboembolic adverse events or serious adverse events were observed between treatment groups.

## Abbreviations

AE = adverse event; AIS = Abbreviated Injury Score; ARDS = acute respiratory distress syndrome; CI = confidence interval; CNS = central nervous system; CT = computed tomography; FFP = fresh frozen plasma; GCS = Glasgow Coma Scale; ICH = intracerebral hemorrhage; ICP = intracranial pressure; ICU = intensive care unit; MOF = multiorgan failure; RBC = red blood cell; rFVIIa = recombinant activated factor VII; SAE = serious adverse event; TBI = traumatic brain injury; TE = thromboembolic; TF = tissue factor.

## Competing interests

YK, BR, and KDB have received lecture and/or consultancy fees from Novo Nordisk A/S (Bagsværd, Denmark). RR has received lecture and/or consultancy fees from Novo Nordisk A/S and has received lecture sponsorship from Novo Nordisk A/S. SBR has received lecture and/or consultancy fees from Novo Nordisk A/S and is a member of the Scientific Advisory Board for rFVIIa. MT is an employee of Novo Nordisk A/S. BW and PITC declare that they have no competing interests. Novo Nordisk A/S is financing the article-processing charge.

## Authors' contributions

All authors made substantive intellectual contributions to the preparation of this manuscript. YK, BR, RR, SBR, KDB, PITC, and BW were co-principal investigators in the original Randomized Control Trial, made substantial contributions to the conception and design of the study and to the analysis and interpretation of data, and were involved in drafting the manuscript and revising it critically. MT made substantial contributions to the conception and design of the study and to the analysis and interpretation of data and was involved in drafting the manuscript and revising it critically. All authors read and approved the final manuscript.
